# Integrated metabolome analysis reveals novel connections between maternal fecal metabolome and the neonatal blood metabolome in women with gestational diabetes mellitus

**DOI:** 10.1038/s41598-020-60540-2

**Published:** 2020-02-27

**Authors:** Chunchao Zhao, Jun Ge, Xia Li, Ruifen Jiao, Yuan Li, Huili Quan, Jianguo Li, Qing Guo, Wenju Wang

**Affiliations:** 1 Shijiazhuang Obstetrics and Gynecology Hospital, Shijiazhuang, 050000 PR China; 2 Institutes of Biomedical Sciences, Shanxi University, Taiyuan, 030006 PR China

**Keywords:** Metabolomics, Metabolic disorders

## Abstract

Gestational Diabetes Mellitus (GDM), which is correlated with changes in the gut microbiota, is a risk factor for neonatal inborn errors of metabolism (IEMs). Maternal hyperglycemia exerts epigenetic effects on genes that encode IEM-associated enzymes, resulting in changes in the neonatal blood metabolome. However, the relationship between maternal gut microbiota and the neonatal blood metabolome remains poorly understood. This study aimed at understanding the connections between maternal gut microbiota and the neonatal blood metabolome in GDM. 1H-NMR-based untargeted metabolomics was performed on maternal fecal samples and targeted metabolomics on the matched neonatal dry blood spots from a cohort of 40 pregnant women, including 22 with GDM and 18 controls. Multi-omic association methods (including Co-Inertia Analysis and Procrustes Analysis) were applied to investigate the relationship between maternal fecal metabolome and the neonatal blood metabolome. Both maternal fecal metabolome and the matched neonatal blood metabolome could be separated along the vector of maternal hyperglycemia. A close relationship between the maternal and neonatal metabolomes was observed by multi-omic association approaches. Twelve out of thirty-two maternal fecal metabolites with altered abundances from 872 1H- NMR features (Bonferroni-*adjusted P* < 0.05) in women with GDM and the controls were identified, among which 8 metabolites contribute (*P* < 0.05 in a 999-step permutation test) to the close connection between maternal and the neonatal metabolomes in GDM. Four of these eight maternal fecal metabolites, including lysine, putrescine, guanidinoacetate, and hexadecanedioate, were negatively associated (Spearman rank correlation, *coefficient value* < −0.6, *P* < 0.05) with maternal hyperglycemia. Biotin metabolism was enriched (Bonferroni-*adjusted P* < 0.05 in the hypergeometric test) with the four-hyperglycemia associated fecal metabolites. The results of this study suggested that maternal fecal metabolites contribute to the connections between maternal fecal metabolome and the neonatal blood metabolome and may further affect the risk of IEMs.

## Introduction

Gestational diabetes mellitus (GDM) has attracted worldwide public health concern due to its adverse maternal, fetal and neonatal outcomes. GDM is a serious pregnancy complication with various risk factors^1^, including overweight, obesity, a family history of diabetes, advanced maternal age, etc. GDM has been linked with an increased risk of inborn errors of metabolism (IEMs) in offspring^2,3^. IEMs are caused by inherited genetic defects and can be influenced by environmental stimuli^4^. Accumulating evidence suggests that maternal hyperglycemia is associated with epigenetic changes in affected offspring^2,5,6^. The transfer of excess glucose across the placenta stimulates fetal pancreatic insulin secretion and results in epigenetic changes to fetal genes involved in metabolic programming, which may contribute to the development of IEMs^2^.

GDM has also recently been associated with changes in the gut microbiota compared to normoglycemic pregnant women^7–9^, and is believed to alter the microbiota of both pregnant women and the neonates^10^. However, although an association between gut microbiota and GDM has been established, the interactions between GDM and the gut microbiota are not yet fully clear^8^.

Metabolites are key players in the interactions between gut microbiota and the host^11^. Short chain fatty acids and trimethylamine oxide produced by gut microbiota play essential roles in host energy metabolism and cardiovascular functions, respectively^12,13^. For this reason, metabolomics has emerged as a promising approach in elucidating the relationship between gut microbiota and the host^14,15^.

Because gestational hyperglycemia has been associated with increased risk of IEMs (featured by metabolic disorders), and maternal gut microbiota is proved to be a contributor of hyperglycemia, we hypothesized that GDM related changes of maternal gut microbiota contributes to the neonatal IEMs. To assess possible connections between maternal gut microbiota and the neonatal IEM-related metabolic disorders, we applied multi-omic association approaches to investigated the relationship between maternal fecal metabolome and the neonatal blood metabolome (Fig. S1). We observed that maternal hyperglycemia is a discriminating factor for the two metabolomes. We further identified maternal fecal metabolites that are responsible for the variations of neonatal blood metabolome between GDM and the healthy control, and provide a discussion of the potential underlying connections.

## Materials and Methods

### Subjects

A cohort of 40 pregnant women who entered Shijiazhuang Obstetrics and Gynecology Hospital (Hebei, China) between March and October of 2018 were enrolled in this study. This cohort included 22 patients with GDM (diagnosed as described below) and 18 NDM controls. Women with pre-existing diabetes, impaired fasting glucose, chronic or serious acute infections, cardiovascular or hematological diseases, or abnormal liver or kidney function were excluded from the cohort. During the 24^th^–28^th^ week of gestation, fasting plasma was collected and a 75-g, 3-hour oral glucose tolerance test (OGTT) was performed. Fasting plasma glucose was measured using an AU5800 Automatic Biochemical Analysis System (Beckman coulter, Brea, CA, USA). GDM was diagnosed according to the criteria of the International Association of Diabetes and Pregnancy Study Group (IADPSG)^16^, with at least one plasma glucose level being no less than the following thresholds: fasting, 5.1 mmol/L, OGTT- 1 hour, 10.0 mmol/L, OGTT- 2 hour, 8.5 mmol/L. The study was conducted according to the guidelines in the Declaration of Helsinki and approved by the Ethics Committee of Shijiazhuang Obstetrics and Gynecology Hospital.

### Demographic data and sample collection

Maternal demographic data were obtained by interview on the day of sample collection, including nationality, parity, age, height, blood pressure, and body weight. Overnight fasting stools were collected from the enrolled pregnant women during the fourth trimester of pregnancy and stored at −80 °C. Dried blood spot samples from the offspring of the enrolled pregnant women were collected by heel stick, spotted on Whatman 903 filter paper sampling cards, air-dried for 3 hours, stored at 2–8 °C, and detected within 24 hours after collection.

### 1H-NMR based untargeted metabolic profiling

Stool samples were prepared for 1H-NMR spectrometry as described previously^17^, with some adjustments. Briefly, 100 mg fecal sample was resolved with 1 ml D_2_O (containing 0.05% TSP (3-trimethylsilyl-[2,2,3,3-D4]-propionate) as internal standard), homogenized in an ice-water bath with an IKA T10 Basic ULTRA-TURRAX disperser (IKA, Germany), and centrifuged at 4 °C, 13, 000 rpm for 20 min. Six hundred microliters of the supernatant was transferred into a 5 mm NMR tube for analysis. 1H-NMR spectrometry was performed using a Bruker 600-MHz AVANCE III NMR spectrometer (Bruker BioSpin, Germany). 1H-NMR spectra were acquired using the noesygppr1d pulse sequence with the following parameters: 64 scans; spectral size, 65536 points; spectral width, 12345.7 Hz; pulse width, 40.5 μs; and relaxation delay, 1.0 s. MestReNova (v8.0.1, MestreLab Research, Santiago de Compostella, Spain) was used for spectra processing. The phase and baseline were corrected manually, and the chemical shift of TSP was calibrated at 0.00 ppm. The spectral region of δ 0.16 to δ 9.58 was segmented to 0.01 ppm widths after excluding the region corresponding to residual water (δ 4.60–δ 5.15). The resulting NMR data was normalized to the total sum of spectra before further analysis.

### Multivariate pattern recognition analysis

SIMCA-P (v14.1, Umetrics AB, Umea, Sweden) was used for multivariate pattern recognition analysis of the 1H-NMR data. Principle Component Analysis (PCA) was performed to maximize the difference between samples and to exclude outliers. Orthogonal Projection to Latent Structures Discriminant Analysis (OPLS-DA), incorporating known classification information, was performed to observe the 1H-NMR features with discriminating power between GDM and the NDM control. The 1H-NMR data was scaled by auto-scaling and pareto scaling for PCA and OPLS-DA, respectively. The best-fitted OPLS-DA model was selected by a cross-validation of all models using a 200-step permutation test. The fitting validity and predictive ability of the selected OPLS-DA model were assessed by the parameters R2Y and Q2, respectively. Differential metabolites were defined as metabolites with altered between-group abundances, and simultaneously meet the following criteria: Importance for the Projection (VIP) values greater than 1 in the selected OPLS-DA model and false discovery rate (*fdr*)-adjusted *P* < 0.05 in an independent-sample t-test. Metabolite enrichment and pathway analysis was performed using the pathway analysis module implemented in MetaboAnalyst web portal (http://www.metaboanalyst.ca).

### Targeted metabolomics of neonatal dry blood spot

Targeted metabolomic profiling of dry blood spots (DBSs) was carried out according to a previously reported protocol^18^ with some adjustments. Briefly, each DBS sample was punched into a 96-well plate, and metabolites were extracted using internal standard containing extraction solvent (50% MeOH, 50% ACN, 250 nM internal standard). The extracted samples were derivatized with 3 N butanolic-HCl at 65 °C, and then reconstituted with an acetonitrile/water (70:30) solution containing 0.05% formic acid. A 10 μl aliquot of each sample was injected to an API 3200 ESI-MS/MS mass spectrometer (Applied Biosystems, USA) for analysis. A total of 42 metabolites (31 acylcarnitines and 11 amino acids, Table S1) were scanned with MRM mode. Each analyte was quantified using the signal intensity ratio of the compound to its internal standard.

### Statistical analysis

Co-Inertia Analysis (CIA) was performed by R software (v3.5.0, package vegan) to assess the consistency of maternal fecal metabolome and the matched neonatal blood metabolome. CIA is a multivariate approach identifying trends or co-relationships in multiple datasets containing the same or matched samples. The parameter RV coefficient (scale 0–1) was applied to assess the global similarity between the two datasets

Procrustes Analysis (PA) was performed by R software (v3.5.0, package vegan) to assess the structural similarity between maternal fecal metabolome and the matched neonatal blood metabolome. PA is a visualization of a superimposition of the sample coordinates of ordination analysis^19^. The parameter *m*^2^ was applied to assess the dissimilarity of the two datasets.

Redundancy Analysis (RDA) was performed by R software (v3.5.0, package vegan) to investigate the contributions of maternal fecal differential metabolites to the association between maternal fecal metabolome and the neonatal blood metabolome. The fitness of each metabolite to an ordination of RDA was evaluated by envfit test. The parameter squared correlation coefficient (*r*^2^) was applied to assess the fit goodness of a metabolite to the correlation.

Between-group statistical analyses were performed via two-tailed *Student’s t-tests* in SPSS 22.0. *P*-values were adjusted with false discovery rate (*fdr*) correction in R (v3.5.0, package *vegan*), and *adjusted P* < 0.05 were defined as statistically significant.

### Ethical approval

All procedures performed in studies involving human participants were in accordance with the Ethical Standards of the Institutional and/or National Research Committee and with the 1964 Helsinki Declaration and its later amendments or comparable ethical standards, and were approved by the Ethics Committee of Shijiazhuang Obstetrics and Gynecology Hospital.

### Informed consent

Informed consent was obtained from all individual participants included in this study.

## Results

### Maternal hyperglycemia discriminates maternal fecal metabolome and the matched neonatal blood metabolome

**Figure 1:**
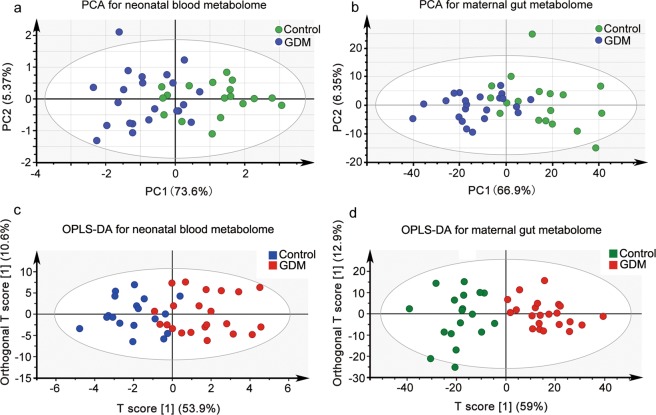
Maternal hyperglycemia discriminates both maternal gut metabolome and the matched neonatal blood metabolome. **(a)** PCA score plot for the neonatal blood metabolome based on targeted metabolic profiling of 42 metabolites (detailed in Table S1) from dry blood spots. The first two principal components (PCs) explained 73.6% and 5.37% of the total variances, respectively. (**b**) PCA score plot for the maternal fecal metabolome based on untargeted metabolic profiling of maternal feces. The first two PCs explained 66.9% and 6.35% of the total variances, respectively. (**c**) OPLS-DA score plot of the neonatal blood metabolome. The horizontal axis represents the predicted score of the first component, which explained 53.9% of the between group variations. The vertical axis represents the orthogonal principal component score, which explained 10.6% of the within-group variations. R2X = 0.461, R2Y = 0.787, Q2 = 0.533. (**d**) OPLS-DA score plot of the maternal gut metabolome based on untargeted metabolic profile from feces. The horizontal axis represents the predicted score of the first component, which explained 59% of between group variations. The vertical axis represents the orthogonal principal component score, which explained 12.9% of the within-group variations. R2X = 0.719, R2Y = 0.791, Q2 = 0.658.

To investigate the effects of maternal hyperglycemia on the neonatal blood metabolome, we compared the DBS metabolome of neonates from mothers with GDM to those from the NDM controls. The DBS metabolome showed a separation trends between the offspring of GDM and the NDM control in PCA score scatter plot (Fig. 1a), and a clear separation in OPLS-DA score scatter plot (Fig. 1c, the OPLS-DA model was validated by a permutation test showed in Fig. S2b). Because plasma metabolome is significantly affected by the gut microbiota^20,21^, we further examined whether hyperglycemia could differentiate the fecal metabolomes between GDM and the NDM controls. From the score scatter plot of PCA (Fig. 1b) and OPLS-DA (Fig. 1d, the OPLS-DA model validated by a permutation test in Fig. S2a) of the offspring-matched maternal fecal metabolome, pregnant women with GDM were clearly separated from the NDM controls. These results suggest that maternal hyperglycemia is a discriminating factor of both the maternal fecal metabolome and the neonatal blood metabolome.

### Maternal fecal metabolome of GDM is associated with the matched neonatal blood metabolome

**Figure 2:**
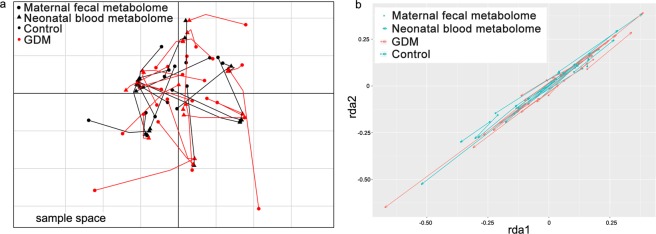
The maternal gut metabolome is closely correlated with the matched neonatal blood metabolome. Correlations between the two metabolomes were assessed by (**a**) Co-Inertia analysis (CIA) of the co-variability and (**b**) Procrustes analysis (PA) of the squared differences. In the sample space of CIA (**a**), solid circle represents maternal fecal metabolome, solid triangle represents the neonatal blood metabolome, samples in black were from the non-diabetic control, samples in red were from GDM. The matched maternal and neonatal samples are linked by edges. The shorter the edge, the better the correlation of the matched samples. In the Procrustes superimposition plot (**b**), solid circle represents maternal fecal metabolome, solid circle with an arrow represents the neonatal blood metabolome. The length of the edge represents the similarity between maternal fecal metabolome and the neonatal blood metabolome. Samples in pink were from GDM, samples in light blue were from the non-diabetic control.

Because the maternal fecal metabolome and the neonatal blood metabolome displayed similar separation trends along the direction of maternal hyperglycemia, we further investigated the potential relationship between these two metabolomes using Co-Inertia Analysis (CIA) and Procrustes Analysis (PA). CIA is a multivariate method that identifies co-variability (trends or co-relationships) in multi-omic datasets that contain the same or matched samples^22^. CIA of maternal fecal metabolome (solid circle) and the matched neonatal blood metabolome (solid triangle) revealed a significant relationship (RV coefficient = 0.72, 999 permutations, *P* < 0.05) between the two datasets (Fig. 2a). Samples from GDM (red) and the healthy control (black) are closely projected, which suggest similar covariations between maternal fecal metabolome and the neonatal blood metabolome of these two groups, indicating the effect of hyperglycemia to the intrinsic communications. PA is a statistical shape analysis used to analyze the distribution of a set of shapes and has been successfully applied to evaluate relationships in multi-omic datasets^23^. Procrustes superimposition of sample coordinates obtained from redundancy analysis revealed a good correlation (dissimilarity parameter *m*^2^ = 0.32) between maternal fecal metabolome and the neonatal blood metabolome. The closely projected samples from GDM (pink) and the healthy control (light blue) in PA (Fig. 2b) demonstrated again the effect of hyperglycemia to the intrinsic communications between maternal fecal metabolome and the neonatal blood metabolome. These results suggest that maternal fecal metabolome is closely correlated with the neonatal blood metabolome.

### Maternal fecal metabolites of GDM contribute to the cluster separation of neonatal blood metabolome

**Figure 3:**
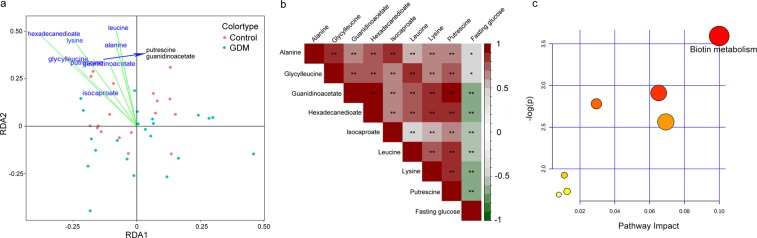
Maternal gut metabolites of GDM contribute to the separation of the matched neonatal blood metabolome. (**a**) Redundancy analysis (RDA) of differential fecal metabolites in the separation of the neonatal blood metabolome. Green dots: samples from the GDM group; red dot: samples from the NDM control group. Envfit test (see Table S2) was performed to evaluate the fitness of each metabolite to the two ordinations in RDA. Only metabolites that significantly correlate (*P* < 0.05) with the separation of neonatal blood metabolome are shown. (**b**) Spearman’s rank correlations between maternal fasting glucose and maternal fecal metabolites that significantly contributed to the separation of neonatal blood metabolome. *Represents *P* < 0.05, **Represents *P* < 0.01. (**c**) Metabolic pathway-enrichment analysis of the metabolites that significantly correlated with maternal fasting glucose (in b) using the MetaboAnalyst web portal. Only pathways with scores of impacts higher than 0.05 and *P-*values lower than 0.05 were labelled (for further details, see Table S4).

To further investigate the extent to which maternal fecal metabolites are associated with changes in the neonatal blood metabolome, we performed redundancy analysis (RDA) using the relative abundance of maternal fecal metabolites (as explanatory variables) and the neonatal DBS metabolome data (as response variables). Prior to RDA, we identified maternal fecal metabolites that had differential relative abundances in the GDM group and the NDM control group. A total of 872 features was obtained in 1H-NMR spectrometry of maternal feces, from which 32 metabolites were assigned. Twelve metabolites with altered abundances (VIP > 1, adjusted *P* < 0.05) between GDM and the NDM control were observed (Table 1). To determine the maternal fecal metabolites that affecting the neonatal blood metabolome, RDA (Fig. 3a) was performed on the DBS metabolome of GDM and the NDM controls with the twelve altered maternal metabolites as environmental factors. The results of RDA revealed a clear cluster separation of the DBS metabolome between GDM and the NDM controls along the vectors of 8 maternal fecal metabolites with high correlation coefficient and strong statistical significance (envfit test, *r*^2^ > 0.1, *adjusted P* < 0.05 under 999 permutations, Table S2). The eight maternal fecal metabolites with a discriminating power to the DBS metabolome between GDM and the NDM control included hexadecanedioate, lysine, leucine, alanine, glycyl-leucine, putrescine, guanidinoacetate, and isocaproate. These results suggested that changes in the abundance of maternal fecal metabolites in GDM contribute to variations of the neonatal blood metabolome.

**Table 1:** Identified fecal metabolites that differed in abundance between GDM and healthy control.

Metabolites	Chemical shift	VIP*	*P*-Value	Adjusted *P*-value	HMDB
ethylmalonate	0.89037	2.22	1.58E-03	1.72E-03	HMDB0000622
adipate	0.92040	2.03	1.07E-04	1.82E-04	HMDB0000448
leucine	0.96046	2.32	7.08E-06	1.74E-05	HMDB0000687
alanine	1.48113	2.34	4.08E-06	1.63E-05	HMDB0000161
glycylleucine	1.59128	1.74	2.20E-04	2.64E-04	HMDB0000759
putrescine	1.72145	1.81	1.01E-05	2.02E-05	HMDB0001414
lysine	1.91169	1.64	1.22E-04	1.82E-04	HMDB0000182
hexadecanedioate	2.20207	1.88	9.05E-03	9.05E-03	HMDB0000672
isocaproate	2.24212	1.51	1.84E-04	2.46E-04	HMDB0000689
N-acetylneuraminate	3.62392	1.57	2.73E-08	3.60E-07	HMDB0000230
guanidinoacetate	3.77411	1.94	7.25E-06	1.74E-05	HMDB0000128
creatine	3.91430	1.66	1.63E-06	9.78E-06	HMDB0000064

*VIP: Variable Importance in the Projection.

### Maternal fecal biotin metabolism of GDM is associated with the neonatal blood metabolome

To further evaluate the correlation between the eight maternal fecal metabolites that contribute to changes in the neonatal blood metabolome, we carried out Spearman rank-correlation analysis (Fig. 3b). Four out of the eight maternal fecal metabolites (guanidinoacetate, hexadecanedioate, lysine, and putrescine) were strongly negatively correlated with maternal hyperglycemia (ρ <−0.60, *P* < 0.05, Table S3). The other four maternal fecal metabolites (alanine, glycyl-leucine, isocaproate, and leucine) showed weak negative correlation with maternal hyperglycemia (−0.6 < ρ < 0, *P* < 0.05, Table S3). Because the hyperglycemia correlated maternal fecal metabolites showed strong positive correlations between each other, we hypothesized that these metabolites share common metabolic pathways. Using pathway enrichment analysis, we found that biotin metabolism was significantly enriched with these metabolites (pathway impact >0.1 adjusted *P* < 0.05, Fig. 3c, Table S4). These results suggested that biotin metabolism in the maternal gut metabolome contributes to maternal hyperglycemia and changes in the neonatal blood metabolome.

## Discussion

In the present study, maternal fecal metabolome and the matched neonatal blood metabolome could be separated along the vector of maternal hyperglycemia. Our multi-omic association studies revealed close relationships between these two metabolomes. We further identified a panel of four maternal fecal metabolites that contribute to the separation of normal and hyperglycemic neonatal blood metabolomes. Pathway analysis of the four closely correlated metabolites suggested that biotin metabolism in the maternal gut may play a role in changes of the neonatal blood metabolome.

### Changes in gut microbiota has been associated with GDM

A distinct microbiota profile is present in patients with GDM^24^, and the ratio of GDM-enriched bacteria to control-enriched bacteria is positively correlated with blood glucose level^9^. Furthermore, members of the gut microbiota are emerging as potential GDM biomarkers^10^. Although such associations between gut microbiota and GDM have been established, the underlying mechanisms by which the microbiota and the host interact remain largely unknown^25^. Metabolites represent some of the key players in the interactions between gut microbiota and the host^12^. Metabolomics therefore provides a powerful tool for investigating the differentially enriched metabolites between study groups^26^. In the present study, we observed a total of 12 maternal fecal metabolites that were differentially enriched between GDM and the NDM controls, among which 4 metabolites (lysine, putrescine, guanidinoacetate, and hexadecanedioate) were responsible for the separation of the mother-matched neonatal blood metabolome. These four metabolites have all been previously reported to be associated with GDM or other diabetes. Plasma lysine level during pregnancy is an independent risk factor for insulin resistance and GDM^27^. Serum putrescine level is significantly correlated with the level of glycosylated hemoglobin^28^. Putrescine partially prevents the dysmorphogenic effects of high glucose in rat embryos^29^. Guanidinoacetate was observed to be significantly decreased in the serum, urinary tract, and renal cortex of diabetic rats^30^. Hexadecanedioate is causally associated with increased blood pressure^31^, increased risk of heart failure and stroke^32^. SNPs at Locus CYP4A11 and SLCO1B1 are associated with the abundance of hexadecanedioate^33^. Biotin metabolism was enriched with the four hyperglycemia associated fecal metabolites in the present study, which has been previously reported to play an essential role in influencing GDM^34^. Combined with previous reports, the results of this study suggest that fecal metabolites (lysine, putrescine, guanidinoacetate, and hexadecanedioate) and biotin metabolism may contribute to gestational hyperglycemia.

### Maternal factors affecting Inborn errors of metabolism (IEMs) of the neonates

IEMs are a large group of rare genetic diseases that result from one of several defects in an enzyme or transport protein that affects a particular metabolic pathway^35^. Although genetic defects are the major determining factors in the occurrence of IEMs^36^, it is believed that maternal environmental factors (such as gestational hyperglycemia) play an nonnegligible role in the epigenetic regulation of gene expression in offspring^2,4^. Maternal GDM is associated with genome-wide DNA methylation changes in the placenta and cord blood of the exposed offspring^2^. Further studies have revealed that methylation at multiple genes/loci regulated by maternal GDM is responsible for the transmission of GDM effects to the next generation^37^. Another study revealed that GDM has epigenetic effects on genes that are preferentially involved in metabolic disease pathways, with consequences to fetal development^2^. Thus, maternal GDM represents a risk factor for IEMs and abnormal fetal development in the exposed offspring^36^, and factors that influence GDM could also affect the risk of IEMs^35^. Four maternal fecal metabolites (lysine, putrescine, guanidinoacetate, and hexadecanedioate) were observed to be correlated with GDM in the present study, which may exert potential effects to the neonatal risk of IEMs. The four maternal fecal metabolites were also correlated with the neonatal IEMs related blood metabolome in this study. All of the four metabolites and their related biotin metabolism have been previously reported to be associated with IEMs. Inborn errors in the metabolism of lysine result in Glutaric Aciduria type 1 (GA1), an inborn error of metabolism that is caused by mutations in GCDH, which encodes glutaryl-CoA dehydrogenase^38^. Brain accumulation of guanidinoacetate can cause developmental delay, seizures, and movement disorders^39^. The accumulation of putrescine is associated with another inborn error of metabolism, cystinuria^40^. The concentration of hexadecanedioate is elevated in the Peroxisome Biogenesis Disorders-Zellweger Syndrome Spectrum disorders^41^. Biotinidase deficiency is an inborn error of metabolism that affects the endogenous recycling of biotin, causing neurological and cutaneous symptoms^42^.

## Conclusions

In the present study, we observed a close relationship between the maternal fecal metabolome and the matched neonatal blood metabolome in GDM. The four maternal fecal metabolites (lysine, putrescine, guanidinoacetate, and hexadecanedioate) that are responsible for the separation of neonatal blood metabolome from GDM and the NDM control have previously been associated with both maternal hyperglycemia and the neonatal inborn errors of metabolism. We therefore conclude that maternal GDM related fecal metabolites are correlated with the neonatal IEMs related blood metabolome. Hyperglycemia-mediated epigenetic regulation or substrate effects of IEMs-related enzyme deficiency are two possible linkers of the correlation.

## Supplementary Information

Supplementary Figures

Supplementary Table S1

Supplementary Table S2

Supplementary Table S3

Supplementary Table S4

## Data Availability

The Raw maternal fecal metabolome has been deposited in MetaboLights with ID MTBLS1248. The neonatal blood metabolome data is listed in Table S1. Codes for CIA, PA and CCA can be found in the help file of each R package. Default settings were used for all software analyses unless otherwise stated.
