# Supplementary material for: Integrated metabolome analysis reveals novel connections between maternal fecal metabolome and the neonatal blood metabolome in women with gestational diabetes mellitus
Source: Sci Rep. 2020 Feb 27;10:3660. doi: 10.1038/s41598-020-60540-2 (PMC7046769; doi:10.1038/s41598-020-60540-2)
Supplement: Supplementary file 1 — Supplementary Figures [file 41598_2020_60540_MOESM1_ESM.doc]

**Integrated metabolome analysis reveals novel connections between maternal fecal metabolome and the neonatal blood metabolome in women with gestational diabetes mellitus.**

**Chunchao Zhao^1, #^, Jun Ge^1, #^, Xia Li^1^, Ruifen Jiao^1^, Yuan Li^1^, Huili Quan^1^, Jianguo Li^2^, *, Qing Guo ^1^, *, Wenju Wang ^1^, ***

1. Shijiazhuang Obstetrics and Gynecology Hospital, Shijiazhuang 050000, PR China

2. Institutes of Biomedical Sciences, Shanxi University, Taiyuan 030006, PR China

# These authors contributed equally to this work.

*** Corresponding author:**

**Professor Wenju Wang**,

Shijiazhuang Obstetrics and Gynecology Hospital, No.206, Zhongshandong Road, Changan District, Shijiazhuang 050000, Hebei, China

Tel/Fax: +86-0311-89291615; Email: wangwjsjz@126.com

**#Co-corresponding author:**

Professor. Qing Guo,

Shijiazhuang Obstetrics and Gynecology Hospital, No.206, Zhongshandong Road, Changan District, Shijiazhuang 050000, Hebei, China

Tel/Fax: +86-0311-89291615; Email: guoqingyz2019@163.com

**Co-Corresponding author**

Dr. Jianguo Li

Key Laboratory of Chemical Biology and Molecular Engineering of Ministry of Education, Shanxi University, No. 92, Wucheng Road, Xiaodian District,

Taiyuan 030006, Shanxi, China

Tel/Fax: +86-351-7018958; Email: lijg@sxu.edu.cn

**Running title:** Maternal fecal metabolome is associated with the neonatal blood metabolome in GDM.

**Figure S1. Flowchart of the study design.** The design of this study from case enrollment, metabolomic profiling, multi-omic association to spearman rank correlation and pathway analysis of the key maternal fecal metabolites responsible for the connections between maternal fecal metabolome and the neonatal blood metabolome.

**
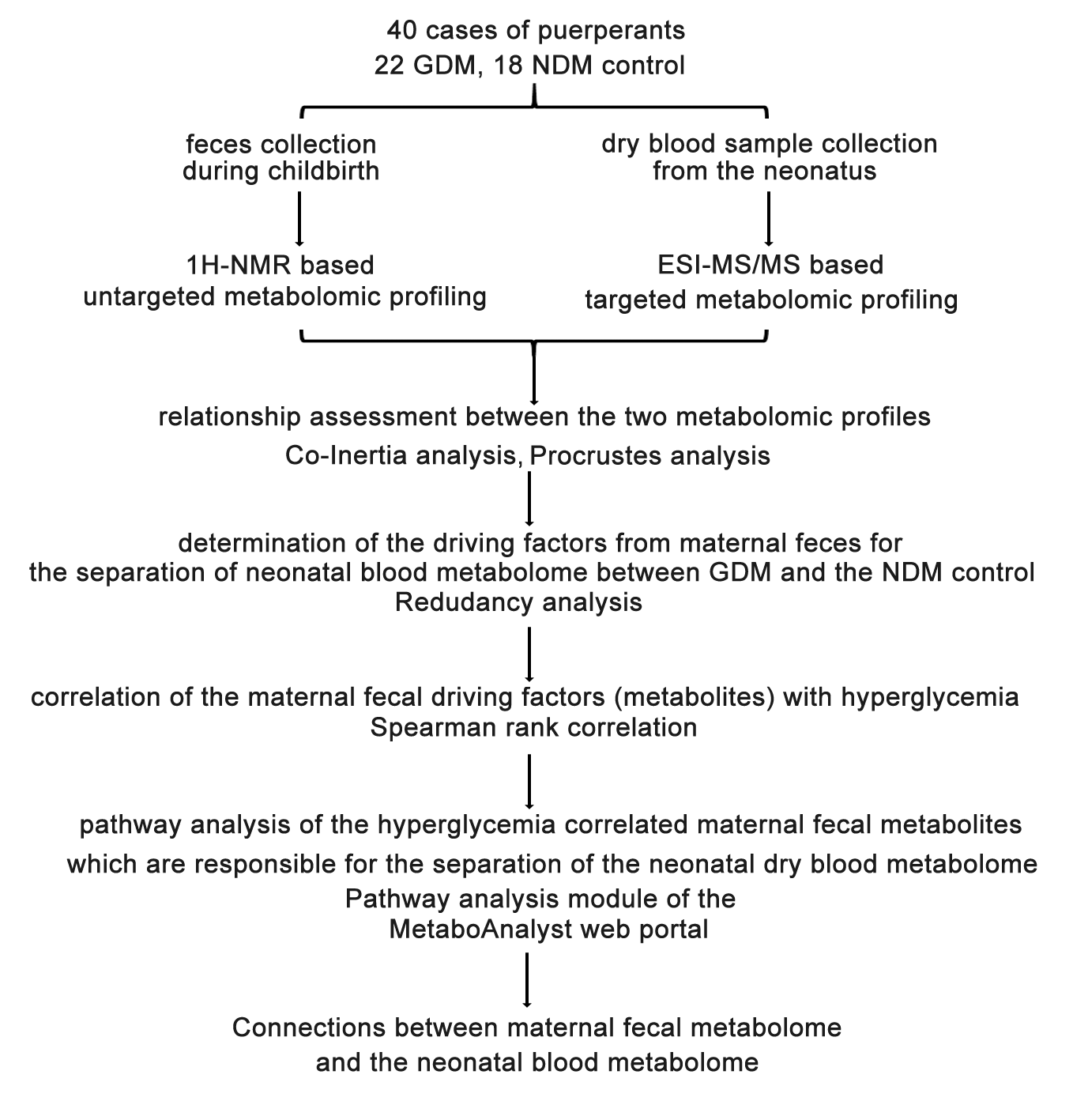
**

**Figure S2. Validation of the selected OPLS-DA models in Figure 1.** The selected OPLS-DA models were validated by permutation tests based on metabolic profiling of (a) maternal gut metabolome and (b) the neonatal blood metabolome with 200 random permutations. The Q2 intercept represents the validation score of the OPLS-DA model; the more negative the Q2 intercept, the more valid the model.

**
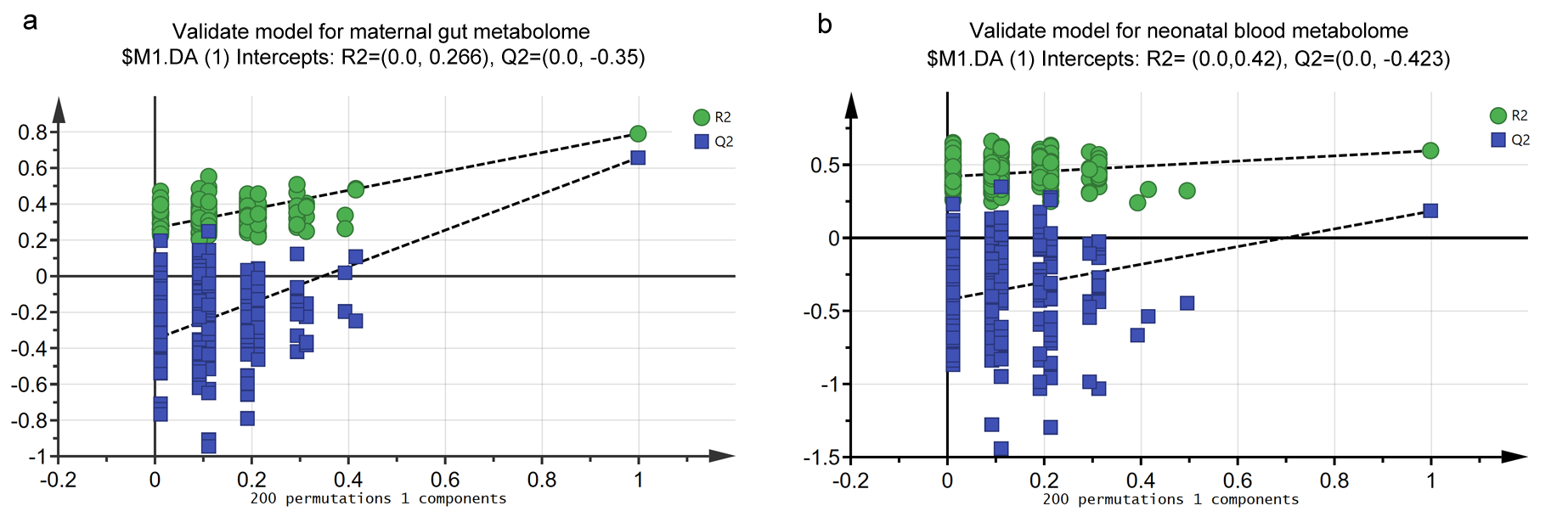
**
